# Shared Governance for Effective Clinical Education in Medical Radiation Science: A Narrative Review

**DOI:** 10.1002/jmrs.70138

**Published:** 2026-10-14

**Authors:** Jacob Leonard Ago, Bismark Ofori‐Manteaw, Minh Chau, Theophilus N. Akudjedu, Yobelli Jimenez, Elio Arruzza, Kelly Bentley‐Spuur

**Affiliations:** ^1^ Faculty of Science & Health School of Dentistry & Medical Sciences, Charles Sturt University Port Macquarie New South Wales Australia; ^2^ Discipline of Medical Radiations School of Health and Biomedical Sciences, RMIT University Bundoora Victoria Australia; ^3^ Teaching Innovation in Radiography Group Charles Sturt University Wagga Wagga New South Wales Australia; ^4^ Department of Medical Imaging Technology, Faculty of Applied Sciences Accra Technical University Accra Ghana; ^5^ School of Dentistry and Medical Sciences, Charles Sturt University Wagga Wagga New South Wales Australia; ^6^ College of Health Sciences, VinUniversity Ha Noi Vietnam; ^7^ Faculty of Health, Environment & Medical Sciences Institute of Medical Imaging & Visualisation, School of Allied Health & Exercise Sciences, Bournemouth University Poole UK; ^8^ Discipline of Medical Imaging Science, Faculty of Medicine and Health University of Sydney Sydney New South Wales Australia; ^9^ School of Allied Health & Human Performance, College of Health, Adelaide University Adelaide South Australia Australia

**Keywords:** institutional effectiveness, medical radiation science education, regulatory accountability, shared governance

## Abstract

Medical radiation science (MRS) education operates within a rapidly evolving healthcare environment characterised by technological advancement, workforce pressures, and increasing regulatory oversight. Effective governance structures are therefore required to ensure that educational programs remain clinically relevant, compliant with accreditation standards, and responsive to students' wellbeing and patient safety imperatives. This review examines the role of shared governance in facilitating institutional effectiveness, regulatory alignment, and sustainable workforce and clinical education. Literature addressing governance theory, MRS education, clinical supervision, accreditation standards, and professional regulation was prioritised in this review. Twenty‐four sources were included and organised across three areas: clinical education and workforce sustainability; professional and regulatory standards; and shared‐governance theory. The literature rarely used the term ‘shared governance’ explicitly in relation to MRS education. Nevertheless, it consistently described distributed responsibilities, interdependence between universities and clinical services, and the need for stakeholder participation in curriculum review, placement planning, assessment, quality assurance and regulatory alignment. Unique vulnerabilities of medical radiation practice, such as rapid technological advancement and global workforce shortages, necessitate agile and inclusive governance structure, especially in settings without clearly outlined governance framework. The review proposes a tri‐integrated model linking academic, clinical, and professional/regulatory governance, with students participating across all three domains. This model offers a practical basis for clarifying roles, improving university–health service coordination, supporting sustainable clinical placements, and maintaining educational quality and patient safety.

## Graphical abstract

This narrative review proposes a tri‐integrated governance model for effective clinical education in medical radiation science. This tri‐integrated module connects academic, clinical and professional or regulatory governance, with students positioned as active participants in decision‐making. Meaningful stakeholder participation may strengthen curriculum relevance, clinical placement sustainability, regulatory alignment, patient safety and workforce readiness in medical radiation science education. Figure created in https://BioRender.com.

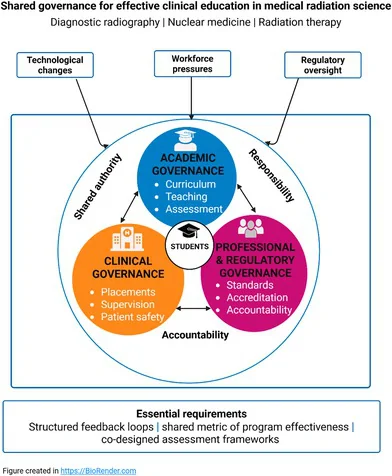

## 1 Introduction

Medical radiation science (MRS) education, encompassing diagnostic radiography/medical imaging, nuclear medicine, and radiation therapy, operates at the intersection of rapid technological advancement, workforce pressures, and increasing regulatory oversight [1, 2, 3, 4]. Contemporary practice is highly dependent on rapidly evolving imaging and therapeutic technologies. Specifically, diagnostic radiography increasingly incorporates artificial intelligence, computed tomography, magnetic resonance imaging, and hybrid imaging; radiation therapy is shaped by image‐guided and adaptive radiotherapy, intensity‐modulated techniques, and automation; and nuclear medicine increasingly uses hybrid systems such as SPECT/CT and PET/CT and expanding theranostic practice [5, 6]. These developments require curricula and clinical education to remain current while supporting safe practice and graduate adaptability [7, 8, 9].

In this review, clinical education refers primarily to the supervised learning undertaken by undergraduate or pre‐registration MRS students during clinical placements. Although diagnostic radiography, radiation therapy, and nuclear medicine students share common challenges such as placement capacity, variable supervision, service pressures, and theory–practice gaps, their clinical education also differs according to professional workflows and technologies. Diagnostic radiography students may rotate through general imaging, emergency, theatre, CT and MRI settings; radiation therapy students learn across treatment planning and treatment delivery pathways; and nuclear medicine students undertake learning involving radiopharmaceuticals, functional and hybrid imaging, and specialised radiation safety. Across the professions, common challenges include limited placement capacity, variable supervision, difficult patient or team interactions, and the need to demonstrate professional capabilities in safety‐critical environments [3, 10, 11]. Although many practitioners value their role in student education, time constraints, burnout, and resource limitations continue to undermine the effectiveness and sustainability of clinical supervision [2, 12].

Given that a substantial proportion of MRS education occurs within clinical settings, these challenges directly affect the quality of student learning and the alignment between academic preparation and clinical practice [2]. While some countries such as Australia and the United Kingdom provide examples of structured accreditation, professional capability and education standards [13, 14, 15, 16], the review is international and not restricted to these jurisdictions. Of note, it remains unclear how governance is structured in many other areas, including low‐ and middle‐income countries [4, 17] despite the distributed responsibility in MRS education between universities, healthcare organisations, professional bodies, accreditation agencies, regulators, clinical educators, and students. Consequently, questions remain regarding how academic, clinical, regulatory, and student stakeholders are represented within governance processes, how decision‐making authority is distributed, and how competing educational, service and regulatory priorities are reconciled.

These governance questions have become increasingly important as MRS programs respond to workforce shortages [3, 18], expanding clinical placement demands [4], evolving accreditation requirements [13] and technological advances [7]. MRS programs must align with accreditation standards, professional capability frameworks, and public safety requirements, ensuring that graduates meet national and international expectations for safe and effective practice [13, 14, 15, 16]. Of note, the MRS profession is transitioning from a purely technical specialty into a socially responsive, community‐embedded healthcare discipline that directly shapes patient outcomes [19] as well as student and workforce wellbeing. The absence of an effective governance structure may contribute to theory‐practice gaps, inconsistencies in clinical learning experiences, workforce misalignment, and difficulties responding to changing professional and healthcare needs. As a result, there is a need for governance structures that not only ensure compliance but also facilitate coordination across academic, clinical and regulatory domains.

In this context, shared governance, defined as a structured, multi‐stakeholder decision‐making model in which authority, responsibility, and accountability are distributed across academic, clinical, and regulatory actors, emerges as a critical mechanism for ensuring institutional effectiveness [20]. In simple terms, shared governance describes how the people and organisations responsible for MRS education contribute to relevant decisions rather than decisions being made by the university alone. Structured shared governance may therefore include joint university‐clinical committees, defined roles for placement partners, student representation, agreed feedback pathways, and shared quality indicators. By integrating diverse perspectives (e.g., from practitioners, students and academic staff and educational policy makers), shared governance supports curriculum relevance, strengthens university‐workplace partnerships, and enhances accountability to professional standards and patient outcomes [20].

To date, no review has synthesised the literature on shared governance within the context of MRS education. This narrative review therefore aimed to examine how shared governance may support effective undergraduate or pre‐registration MRS education across university and clinical placement settings. It also sought to identify principles for integrating academic, clinical, professional or regulatory, and student participation within MRS education governance.

## 2 Methods

A narrative review approach was employed. Narrative reviews are useful for examining diverse evidence bases and developing conceptual frameworks through the interpretation of existing literature [21].

### 2.1 Literature Search

Searches were conducted across MEDLINE, CINAHL, Scopus, PubMed via EBSCOhost, supplemented by targeted searches via Google Scholar. Key terms such as “shared governance,” “clinical education,” “institutional effectiveness,” “radiography,” “medical radiation,” “radiation therapy,” “nuclear medicine,” “workforce sustainability” and “health practitioner regulation” were included in the search strategy. These terms were incorporated into the search strategy and combined using Boolean operators (AND/OR) to enhance search sensitivity. The search was not restricted by a starting publication year and included English language literature published up to February 2026.

Peer‐reviewed empirical studies, review articles, conceptual papers and relevant professional, accreditation and regulatory documents were considered where they linked governance principles, such as accountability, autonomy and transparency, to institutional or program outcomes, teaching and learning, clinical education, or professional standards. MRS‐specific regulatory frameworks and professional body documents were also analysed to provide contextual relevance and justification for the proposed governance model. Opinion pieces without a substantive conceptual contribution and non‐English language publications were excluded.

## 3 Results

Twenty‐four articles and documents were included in this review. The included literature comprised peer‐reviewed empirical studies, review and conceptual articles, and professional, accreditation, and/or regulatory documents. The included studies originated from Europe (*n* = 5), United States of America (*n* = 1), the United Kingdom (*n* = 2), Africa (*n* = 3), Australia (*n* = 4), and global perspective (*n* = 9). Most of the included studies were review‐type articles (*n* = 10), followed by quantitative studies (*n* = 5), regulatory documents (*n* = 5), qualitative studies (*n* = 3), and mixed‐method research (*n* = 1). As a narrative review, the included literature is not exhaustive. For instance, a more targeted and comprehensive examination of regulatory, accreditation, and professional standards could enable a comparative analysis of similarities and variability in governance approaches across regions, particularly between high‐income countries and low‐ and middle‐income settings.

For clarity, the evidence included was organised into three focus areas: (1) clinical education and workforce sustainability; (2) professional, accreditation, and regulatory standards; and (3) shared governance in higher education. Tables 1A, 1B, 1C summarise the included literature according to these focus areas.

**TABLE 1A:** Clinical education and workforce sustainability.

Author(s), year	Country/region	Study type	Focus area	Governance relevance
Munn et al. [1], 2020	International	Discussion paper	Change management and evidence‐based practice	Highlights the need for adaptive educational structures capable of responding to technological and professional change.
England et al. [22], 2017	Europe	Quantitative cross‐sectional study	Clinical radiography education	Demonstrates variability in clinical education models and stakeholder involvement across countries.
Lundvall et al. [9], 2021	Sweden	Qualitative study	Clinical learning during placements	Demonstrates the interdependence of academic and clinical learning environments in professional formation.
Young et al. [23], 2023	Ireland	Quantitative cross‐sectional study	Clinical supervision	Highlights the central role of clinical supervisors in student learning and competency development.
O'Connor et al. [2], 2023	Ireland	Cross‐sectional, mixed‐method study	Clinical learning environment	Identifies factors influencing student experiences in clinical education.
Ofori‐Manteaw et al. [12], 2024	Ghana	Quantitative, cross‐sectional study	Preceptorship and supervision	Highlights challenges facing clinical educators and the need for stronger academic–clinical collaboration.
Ago et al. [24], 2025	Ghana	Qualitative study	Clinical placement experiences	Demonstrates how clinical learning environments influence student learning and professional development.
Bwanga et al. [25], 2025	Zambia	Quantitative study	Audit of clinical education in Zambia	Identifies resource and governance challenges affecting clinical education quality.
Susiku et al. [3], 2024	International	Systematic review	Education models and workforce	Highlights the relationship between educational structures, employability, and workforce sustainability.
Zhu [26], 2024	International	Population‐based workforce study	Workforce planning	Identifies growing workforce demands requiring responsive educational systems.
Shields et al. [27], 2024	Australia	Qualitative study	Burnout	Highlights organisational challenges to workforce sustainability concerns in nuclear medicine.
Chau et al. [18], 2026	Australia	Narrative review	Workforce shortages	Demonstrates the importance of aligning education with workforce planning.
Tay and McNulty [28], 2023	International	Narrative review	Future of radiography education	Discusses contemporary challenges and opportunities shaping radiography education.

**TABLE 1B:** Professional, accreditation, and regulatory standards.

Author(s), year	Country/region	Study type	Focus area	Governance relevance
McNulty et al. [8], 2025	Europe	Guideline document	Radiographer education standards	Evaluates education and training requirements for radiographers across EU Member States and provides recommendations for curriculum development and educational governance.
Susiku et al. [4], 2025	International	Comparative document analysis	Regulation of radiography education	Demonstrates variation in regulatory approaches and professional standards across jurisdictions.
Health and Care Professions Council (HCPC) [16], 2023	United Kingdom	Regulatory standards	Standards of proficiency for radiographers	Defines regulatory expectations for professional competence.
Health and Care Professions Council (HCPC) [15], 2017	United Kingdom	Regulatory standards	Education and training standards	Defines regulatory expectations for educational quality.
Medical Radiation Practice Board of Australia [13], 2019	Australia	Accreditation standards	Accreditation standards	Establishes accreditation and competency requirements for programmes.
Medical Radiation Practice Board of Australia [29], 2026	Australia	Professional practice standards	Professional capability standards	Outlines professional capability requirements for medical radiation practice in Australia.
Joint Review Committee on Education in Radiologic Technology [30], 2002	United States	Accreditation standards	Radiologic sciences education and programme quality assurance	Establishes accreditation requirements for program governance, curriculum design, clinical education, faculty qualifications, student support, assessment, and continuous quality improvement, highlighting the shared responsibility of academic institutions and clinical partners in ensuring educational quality and public protection.

**TABLE 1C:** Shared governance in higher education.

Author(s), year	Country/region	Study type	Focus area	Governance relevance
Trakman [31], 2008	International	Conceptual paper	University governance	Explores representation, authority and accountability in governance systems.
Taylor [32], 2012	International	Conceptual paper	Shared governance	Examines stakeholder participation in university decision‐making.
Stensaker and Vabø [33], 2013	International	Conceptual paper	Shared governance and leadership	Discusses governance adaptation in changing higher education environments.
Raza et al. [34], 2024	International	Systematic review	Shared governance	Demonstrates variability in conceptualisation and implementation of shared governance across higher education.

### 3.1 Clinical Education and Workforce Sustainability

Many of the included studies focus on MRS education and clinical learning environments (CLEs), highlighting the central role of clinical placements, supervision, and professional socialisation in shaping students' professional readiness [1, 2, 8, 9, 12, 22, 23, 25, 28, 35]. The literature also identified workforce and sustainability challenges, including global shortages of medical radiation practitioners, difficulty in recruiting and retaining clinical educators, and high levels of burnout [2, 12, 18, 26, 27]. These challenges threaten the sustainability of clinical education systems and underscore the importance of governance structures capable of aligning educational priorities with evolving workforce and service demands, particularly in rural and underserved settings [3, 4, 18].

### 3.2 Professional, Accreditation, and Regulatory Standards

Some of the included literature related to regulatory, accreditation, and professional standards, which play a critical role in shaping MRS education. Professional capability standards, accreditation guidelines, and qualification frameworks establish minimum competency requirements, ensure public safety, and influence curriculum design and program delivery [4, 8, 13, 14, 15, 16, 30]. These structures shape workforce readiness and professional adaptability within CLEs characterised by many challenging clinical interactions (CCIs) [35, 36].

### 3.3 Shared Governance in Higher Education

The review also drew on literature examining shared governance in higher education, including theoretical models, faculty participation, and organisational performance [31, 32, 33, 34]. The term shared governance was used explicitly in this higher education literature, but was rarely used in the included MRS literature. In the MRS studies and documents, shared governance was implied through descriptions of distributed responsibility, university‐clinical collaboration, stakeholder participation, and shared accountability for curriculum, placements, professional standards, and educational quality. These findings highlight the limitations of traditional academic governance models and underscore the need for expanded, multi‐stakeholder approaches, forming the basis for the following conceptual discussion.

## 4 Discussion

### 4.1 The Stakeholder Theory Underpinning Shared Governance

Stakeholder theory posits that involving multiple actors, including clinical partners, professional bodies, and students, leads to better‐informed decisions that reflect the complexities of the healthcare environment [31, 37]. The theory emerged from an organisational context where it was believed that the organisation was not self‐sufficient but depended on its internal and external interest groups [37]. In MRS, this suggests that governance dominated by academics with no continuous clinical practice engagement may lack the professional expertise required for evolving clinical practice and for developing students' resilience. However, multi‐actor governance structures may enhance workforce responsiveness by facilitating alignment between educational preparation, evolving clinical practice expectations, and professional capability requirements [3, 37].

Specifically, governance of higher education programs must involve a diverse array of stakeholders including students, teaching and non‐teaching staff, corporate associates, government bodies, and the general public [31]. By extension, stakeholder theory suggests that higher education institutions (HEIs) seeking to optimise institutional effectiveness and prepare students for increasingly complex CLEs should consider the interests, expertise, and interactions of diverse stakeholders within and beyond the university context [31]. In MRS education, such collaboration may strengthen curriculum responsiveness, support the development of sustainable clinical education models, and contribute to the development of a resilient and future‐ready workforce.

### 4.2 Traditional Shared Governance in Higher Education

Shared governance in higher education is traditionally defined as the collective and mutual oversight of university operations partaken equally by faculty and administrators [38, 39]. This model is often viewed as a ‘checks and balances’ system designed to protect academic freedom and ensure internal legitimacy [32, 39]. Historically, two primary cultures have dominated this space: collegial governance and managerial governance [32, 33, 38, 40].

Collegial governance is rooted in a model of representative democracy and emphasises consensus‐building, faculty expertise in curriculum design and the university as a community of scholars [33, 38]. In contrast, managerial governance is driven by ‘managerialism’. This model prioritises economic efficiency, top‐down strategic transformation, and performance metrics, often viewing students as *consumers* and the university as a *corporate enterprise* [32, 33, 40]. These models do not sufficiently account for the complexities of health professions education, such as MRS, where a substantial proportion of training occurs in external clinical environments, and where regulatory compliance directly affects public safety [4]. For this reason, health professions education requires a more explicitly integrated and multi‐stakeholder structure, as described in the next section.

### 4.3 Expanding Governance in MRS Education

MRS education involves a broader stakeholder network, including academic leaders, clinical educators, and supervisors, hospital service managers, professional bodies, statutory regulators, and students. Elements of shared governance reflecting this diversity, such as the inclusion of citizens, health administrators, academic educators, learners, and other related sectors, including health services providers, industry, and non‐governmental organisations and policy makers [41, 42, 43], are well established in some HEIs. However, governance structures do not consistently extend beyond the traditional faculty‐senate binary, particularly in ways that formally align decision‐making authority. Establishing an inclusive governance structure as a multi‐actor network with specific responsibilities will support alignment between decision‐making authority and the distributed responsibilities of academics, clinical practitioners, students, and regulatory stakeholders, thereby enhancing institutional effectiveness in areas such as sustainable MRS clinical education and students' wellbeing development.

#### 4.3.1 Higher Education Institutions (HEIs)

In this review, academic leaders refer to deans, heads of school or department, program or course directors, clinical education coordinators, subject coordinators, and teaching academics. These staff play a key role in ensuring institutional alignment, curriculum responsiveness and sustainable programme delivery [7, 8, 44]. They are responsible for designing and developing the curricula that guide both university‐ and practice‐based learning [45], while ensuring that educational content remains aligned with evolving professional standards, workforce expectations, and contemporary healthcare needs. In addition, academic leaders facilitate regular and systematic auditing of teaching and learning activities, supporting constructive alignment between university and clinical learning outcomes, professional capability requirements, and workforce preparedness [46]. The Society and College of Radiographers (SCoR) [46] suggest that audits should include ‘feedback from the learners and the supervisors [and] proposed enhancements and action plans …’, highlighting the importance of collaborative and continuous quality improvement within MRS education.

#### 4.3.2 Placement Supervisors and Educators

Clinical supervisors and educators serve as ‘front‐line educators’ and provide hands‐on training and socialise students into professional practice [11, 12, 46, 47]. The inclusion of individuals with clinical expertise in the governance of MRS programs is vitally important as clinical placement is fundamental to professional behaviour and attitude development [11, 48]. Aside from teaching students the ‘knowing how to be’ of the profession [49] (i.e., facilitating students' professional identity formation and resilience), placement educators and/or supervisors perform key duties including rostering students, conducting clinical assessments in line with university (and by extension, accreditation) standards, and providing feedback to both students and the university [2, 12, 50]. Indeed, MRS program success depends largely on placement educators and/or supervisors. Their role in the implementation of the curriculum into clinical practice, both in terms of teaching and assessment [2], makes them an essential part of the governance structure of MRS programs. Clinical educators/supervisors also serve as mentors to students and help them to develop resilience during CCIs [51, 52].

To facilitate these roles, clinical educators and/or supervisors have individual responsibilities to ensure that they are familiar with the program curriculum and design, understand the standards and achievements expected at each level of training, and have a clear understanding of expected learning outcomes [46, 53]. Since curriculum provides the framework for teaching and assessment, the perspectives of clinical educators and/or supervisors are particularly important during periods of curriculum revisions to provide insights into how revised intentions can be effectively implemented and how teaching and learning can be improved [54]. However, it appears that these equally important stakeholders are often overlooked in terms of curriculum revisions and the larger scope of governance in MRS education [10].

For example, in a study that examined clinical educators' perspectives on South Africa's transformed radiography curriculum, one participant indicated that ‘… this new curriculum is very complex. It's something that I feel like we should have a proper meeting where we'll come, maybe to the university, and then they'll be able to discuss everything with us …’ [54, p. 6]. The statement highlights the curriculum's structural complexity and the relational disconnection between universities and clinical educators and/or supervisors, who find the curriculum overwhelming and poorly communicated. It also highlights how MRS programs may exclude clinical partners, especially clinical educators and/or supervisors, from curriculum review and accreditation committees. Another critical issue that may hinder the active participation of clinical educators and supervisors in MRS education governance is the lack of tutoring frameworks to support practitioner transition into clinical education and/or supervision roles [49].

#### 4.3.3 Health Service Partners

Health service partners, such as hospital administrators and clinical managers, oversee the physical and human resources where 50% of MRS training occurs [46, 55]. Clinical partners are expected to collaborate with HEIs to maintain good communication and effective feedback between placement centres and HEIs to ensure that both clinical staff and students are clear about the expected learning outcomes, the relationship between theory and practice, and the teaching and assessment criteria within CLEs [46]. Excluding health service partners from MRS program governance may reduce the quality of clinical education and student support as practitioners may not receive the needed support to engage in clinical supervision and/or education. Notably, the literature indicates a lack of protected time for medical radiation practitioners interested in the clinical supervision of students [2], limiting effective clinical supervision and knowledge transfer [12, 23].

#### 4.3.4 Professional and Statutory Regulatory Bodies

Professional bodies are responsible for defining professional capabilities and ethical codes [4, 45]. On the other hand, statutory regulators, such as the Medical Radiation Practice Board of Australia (MRPBA) and the Health and Care Professions Council (HCPC), enforce registration and safety standards [4, 8]. They guide evaluation of the appropriateness of programs in line with accreditation standards, inform standards related to initial and ongoing registration, and assessment of equivalence for registration purposes (e.g., an overseas application) [45]. In addition to professional regulatory bodies, statutory regulatory bodies include institutions in charge of higher education accreditation and standards [56, 57]. These agencies ensure that university subjects are taught at the appropriate level on national qualification frameworks. For example, in Australia, the Tertiary Education Quality and Standards Agency (TEQSA) ensures that MRS programs taught at the Bachelor level correspond to Australian Qualification Framework Level 7 [58]. The roles of these institutions in ensuring that governance of MRS programs produce graduates with current evidence‐based capabilities [7, 30] make them essential stakeholders in the governance of MRS program.

#### 4.3.5 Students

The SCoR require all students to be ‘… conversant with the curriculum of their own program of study, including methods of assessment and the role played by the clinical supervisor in the assessment process’ [46, p. 8]. Students are also expected to provide guidance on the expected depth and range of learning, support self‐monitoring of progress, and define expectations and inform career development plans and professional development portfolios [45]. With these roles in mind, the SCoR recognise students as essential members of MRS program governance [45, 46]. Students should therefore be given a platform to serve as active stakeholders, whose participation in program governance is a *requisite indicator* of educational excellence and institutional accountability [20, 59].

This expanded model differs from traditional faculty senate models because it acknowledges the university‐health service partnership as the primary site of education [55]. While traditional models focus on internal campus policy, expanded MRS governance addresses *street‐level* implementation issues [60] such as placement capacity, clinical mentorship, and the distribution of benefits and risks between academic and clinical sites [47, 55, 61].

### 4.4 How Shared Governance May Support Effective MRS Education

Institutional effectiveness in MRS education must move beyond generic metrics such as student satisfaction or enrolment numbers [37, 40]. In the context of MRS, ‘effectiveness’ is defined by the program's ability to produce safe, competent, and adaptable practitioners through a sustainable and legally compliant framework [3, 8]. This extends beyond graduate output to include the system's ability to support workforce resilience, professional identity formation, and long‐term workforce sustainability [51, 62] in rapidly evolving healthcare environment. Workforce resilience is treated here as the education system's capacity to continue preparing safe and adaptable graduates despite workforce shortages, service pressures, and technological changes. Effectiveness may be measured by factors such as graduate clinical competence, patient safety outcomes, theory‐practice alignment, student resilience and professional identity formation, clinical supervisor satisfaction, sustainability of university‐workplace partnerships, and accreditation compliance (Table 2). By defining effectiveness through these MRS‐specific lenses, governance becomes a mechanism for operationalising shared responsibility and stakeholder participation to facilitate the delivery of sustainable, resilient, and future‐ready healthcare education [3, 63].

**TABLE 2:** Examples of indicators of effective governance in MRS education.

Indicator	Descriptor
Graduate clinical competence	The primary outcome, measured by the graduate's ability to perform multi‐modality tasks (e.g., computed tomography [CT], magnetic resonance imaging [MRI], single‐photon emission computed tomography/CT [SPECT/CT], intensity‐modulated radiation therapy [IMRT]) and navigate complex clinical scenarios independently [3, 8].
Patient safety outcomes	Ensuring that education directly results in a culture of safety, where graduates minimise clinical errors and adhere to strict radiation protection standards [8, 64, 65, 66].
Theory–practice alignment	The success of the ‘curricular return’ loop, where clinical experiences actively inform and transform the academic curriculum rather than existing as a disconnected accident of the programme [3, 61].
Student resilience and professional identity formation	The effectiveness of professional socialisation, fostering a sense of belonging and a commitment to lifelong learning [11, 51, 62].
Clinical supervisor satisfaction	A critical metric for placement sustainability; programs are only effective if supervisors feel supported and adequately trained to teach [46, 67].
Sustainability of university‐workplace partnerships	The health of the three‐way partnership (student–university–workplace), characterised by mutual trust, shared decision‐making and joint problem‐solving [55, 67].
Accreditation compliance	Adherence to national and international standards which serve as a benchmark for professional quality and public confidence [4, 8].

### 4.5 Strategic Pathways for Effective Governance in MRS Education

#### 4.5.1 Curriculum Quality and Relevance

MRS programs should embed strong clinical representation within curriculum committees to ensure alignment between academic content and evolving clinical practice. Employer‐led stakeholder engagement can accelerate curriculum updates and ensure graduates develop the multi‐modality competencies required by the contemporary workforce [3, 10, 68].

#### 4.5.2 Clinical Education and Placement Sustainability

Shared governance reduces friction in university‐workplace learning partnerships [55, 67]. MRS programs and clinical partners should actively integrate clinical supervisors into governance processes to reduce role ambiguity and strengthen university‐workplace learning partnerships. Clear expectations for supervision, assessment, and feedback must be co‐developed to improve student learning experiences and ensure sustainable placement models [11, 55, 67].

### 4.6 Unique Vulnerabilities of MRS Education

MRS education faces a persistent curriculum lag as equipment evolution (e.g., AI and hybrid imaging) outpaces academic approval cycles [3, 8]. Governance must be agile enough to allow for rapid curricular integration of emerging technologies [69]. Unlike generic liberal arts, MRS governance directly affects patient safety and radiation protection [4, 64]. Ineffective clinical supervision can lead to clinical errors and challenging clinical experiences [35], making robust clinical governance a legal and ethical mandate [4, 64].

Moreover, MRS programs are uniquely vulnerable to external regulatory shifts [4, 8]. Changes in national standards of proficiency can threaten existing training models, requiring governance structures that maintain continuous alignment with statutory bodies [4, 7, 8]. Of note, the global shortage of radiographers and resulting burnout affect the ability of clinical staff to participate in shared governance [3, 11]. Programs are vulnerable if they rely on governance service from a workforce that is already overstretched and under‐resourced [2, 47]. Governance structures must therefore be sufficiently agile to accommodate rapid technological change while maintaining alignment with regulatory standards. Institutions should implement mechanisms that enable timely curriculum adaptation, particularly in response to emerging technologies such as AI and hybrid imaging.

### 4.7 Tensions and Risks of Expanding Shared Governance

To mitigate these risks, institutions must clearly define stakeholder roles, ensure equitable participation, and align governance responsibilities with accountability structures. Role ambiguity often arises between university academics and hospital managers regarding who is responsible for specific learning outcomes [47, 55]. There is also a risk of governance fatigue, where the volume of committee work displaces the core activities of teaching and clinical practice [38, 63].

Furthermore, student and staff involvement can become tokenistic if they are invited to the table but lack genuine decision‐making authority [34, 47]. Power imbalances remain a threat; in many university councils, administrators still hold dominant scores in decision‐making dimensions, potentially marginalising some stakeholders such as students and early career practitioners who provide the most critical front‐line insights [20, 44, 47]. Finally, a move towards total consensus can slow decision‐making, making institutions less responsive to external healthcare crises [32, 40].

### 4.8 Towards an Integrated and Proportional Governance Model for MRS Education

Inclusive governance is frequently associated with enhanced legitimacy, improved decision quality, and stronger stakeholder commitment [20]. However, expanding shared governance without proportional alignment between authority and accountability may undermine, rather than enhance, the effectiveness of MRS programs. Literature also indicates that determining which stakeholder should be represented, how they should be represented, and the extent of their authority remain problematic [31, 37].

Consequently, this review proposes a tri‐integrated governance model for MRS education (Figure 1). The model consolidates rather than replaces existing program, clinical, and regulatory structures. Patient safety and social accountability provide its central purpose [34, 61], while decisions are shared across three connected domains: academic, clinical, and professional/regulatory governance.

**FIGURE 1:**
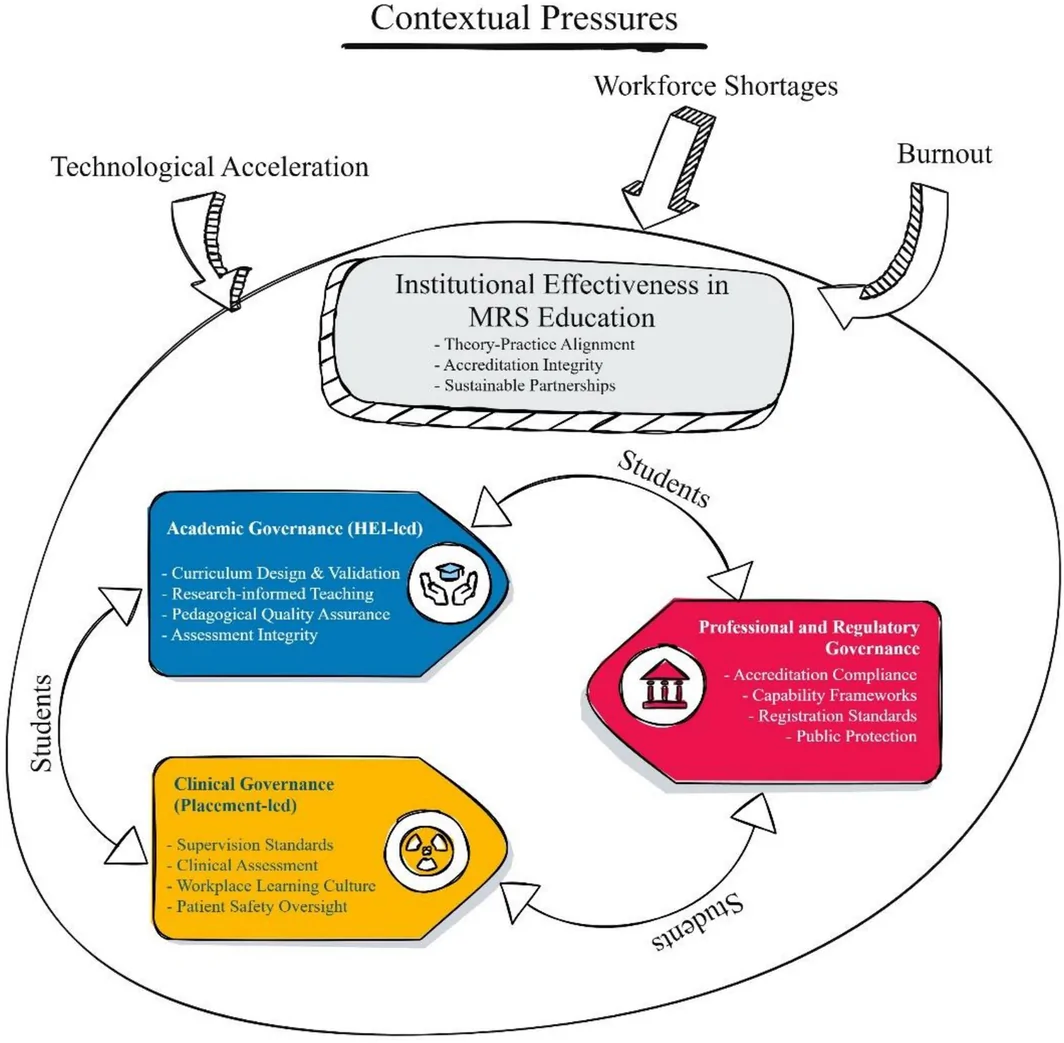
Tri‐integrated governance model for institutional effectiveness in MRS education. The contextual/external pressures are illustrative rather than exhaustive and represent examples of external system pressures influencing governance in MRS education.

This model is underpinned by proportional participation to ensure a careful alignment between representation, authority, and accountability. It comprises three interdependent governance domains. First, academic governance focuses on pedagogical quality, research‐driven learning, and degree integrity [8, 39, 45]. Second, clinical governance addresses placement capacity and safety, supervisor competence, and front‐line service quality [45, 64, 67]. Finally, professional/regulatory governance ensures accreditation compliance, workforce mobility, and threshold competency profiles [4, 8, 45].

These three pillars are connected through structured feedback loops, shared metrics of program effectiveness, and co‐designed assessment frameworks. Importantly, students serve as active participants in governance through their roles in feedback systems, curriculum co‐design, and evaluation of learning and clinical experience [45, 46].

### 4.9 Implications for Education and Practice

Universities offering pre‐registration MRS programs should formalise clinical representation within curriculum, assessment, and clinical education committees to support theory‐practice alignment and minimise drift towards purely academic metrics [3, 61]. This includes establishing structured university‐workplace feedback mechanisms that enable rapid identification of curriculum gaps and align governance cycles with regulatory review timelines. Written terms of reference should specify which decisions are advisory, jointly determined, or retained by the accountable institution. Clinical departments should negotiate protected governance time for clinical supervisors, acknowledging that student supervision, assessment, and governance engagement are statutory and professional obligations [11, 46], support educator development programs, and strengthen communication with academic partners.

Professional bodies, accreditation agencies, and regulators should establish clear communication channels and early guidance regarding evolving competency expectations, particularly for multi‐modality scope and registration [4, 8]. Students should be recognised as essential contributors to education quality, and supported to move beyond symbolic participation to structured participatory roles in program review and development, ensuring their voice is a requisite indicator of quality [20, 59].

## 5 Limitations

It is important to acknowledge that the generalisation of the findings is limited due to the inclusion of only English language literature and the use of a narrative review approach. A comparative analysis of current governance frameworks across different systems is needed to understand geographic differences in shared governance to provide context‐specific best‐practices in line with local professional and regulatory requirements and resource availability.

## 6 Conclusion

Effective pre‐registration MRS education can be achieved by integrating academic leadership, clinical expertise, regulatory oversight, and student voices within a coherent and structured partnership. This inclusive governance structure is essential to bridging the theory‐practice gap and ensuring that graduates are ‘fit for purpose’ in a rapidly evolving healthcare landscape. The integration will also facilitate the development of graduates who are not just work‐ready, but those who are professionally ready to deliver safe, competent, and socially accountable patient care. Nonetheless, participation must be meaningful, structured, and rewarded to avoid the myth of shared governance and ensure that governance remains aligned with educational, workforce, and patient needs. These principles should be adapted to the distinct educational and clinical contexts of diagnostic radiography, radiation therapy, and nuclear medicine.

## Author Contributions

**J.L.A.:** conceptualisation, methodology (literature search), formal analysis, writing – original draft, writing – reviewing and editing. **B.O.‐M.:** conceptualisation, methodology (literature search), writing – reviewing and editing. **M.C.:** conceptualisation, writing – reviewing and editing, visualisation. **T.N.A.:** supervision, writing – reviewing and editing. **Y.J.:** methodology (literature search), writing – reviewing and editing. **E.A.:** methodology (literature search), writing – reviewing and editing. **K.B.‐S.:** supervision, writing – reviewing and editing.

## Funding

The authors have nothing to report.

## Disclosure

*Generative AI Use*: The authors have nothing to report.

*Clinical Trial Registration*: The authors have nothing to report.

## Ethics Statement

Not required for narrative reviews.

## Consent

The authors have nothing to report.

## Conflicts of Interest

M.C. and K.B.‐S. are Associate Editors of *JMRS*. However, as authors of this submission, they had no role in or visibility of the handling of the manuscript through the editorial or peer review process.

## Data Availability

All data (literature) associated with this work have been cited within the paper.
